# Quantitative Analysis of α-L-Iduronidase Expression in Immunocompetent Mice Treated with the *Sleeping Beauty* Transposon System 

**DOI:** 10.1371/journal.pone.0078161

**Published:** 2013-10-21

**Authors:** Elena L. Aronovich, Bryan C. Hall, Jason B. Bell, R. Scott McIvor, Perry B. Hackett

**Affiliations:** Department of Genetics, Cell Biology and Development and the Center for Genome Engineering, University of Minnesota, Minneapolis, Minnesota, United States of America; University of Michigan School of Medicine, United States of America

## Abstract

The *Sleeping Beauty* transposon system, a non-viral, integrating vector that can deliver the alpha-L-iduronidase-encoding gene, is efficient in correcting mucopolysaccharidosis type I in NOD/SCID mice. However, in previous studies we failed to attain reliable long-term alpha-L-iduronidase expression in immunocompetent mice. Here, we focused on achieving sustained high-level expression in immunocompetent C57BL/6 mice. In our standard liver-directed treatment we hydrodynamically infuse mice with plasmids containing a SB transposon-encoding human alpha-L-iduronidase, along with a source of SB transposase. We sought to 1) minimize expression of the therapeutic enzyme in antigen-presenting cells, while avoiding promoter shutdown and gender bias, 2) increase transposition efficiency and 3) improve immunosuppression. By using a liver-specific promoter to drive IDUA expression, the SB100X hyperactive transposase and transient cyclophosphamide immunosuppression we achieved therapeutic-level (>100 wild-type) stabilized expression for 1 year in 50% of C57BL/6 mice. To gain insights into the causes of variability in transgene expression, we quantified the rates of alpha-L-iduronidase activity decay *vis-a-vis* transposition and transgene maintenance using the data obtained in this and previous studies. Our analyses showed that immune responses are the most important variable to control in order to prevent loss of transgene expression. Cumulatively, our results allow transition to pre-clinical studies of SB-mediated alpha-L-iduronidase expression and correction of mucopolysaccharidosis type I in animal models.

## Introduction

**Figure 1:**
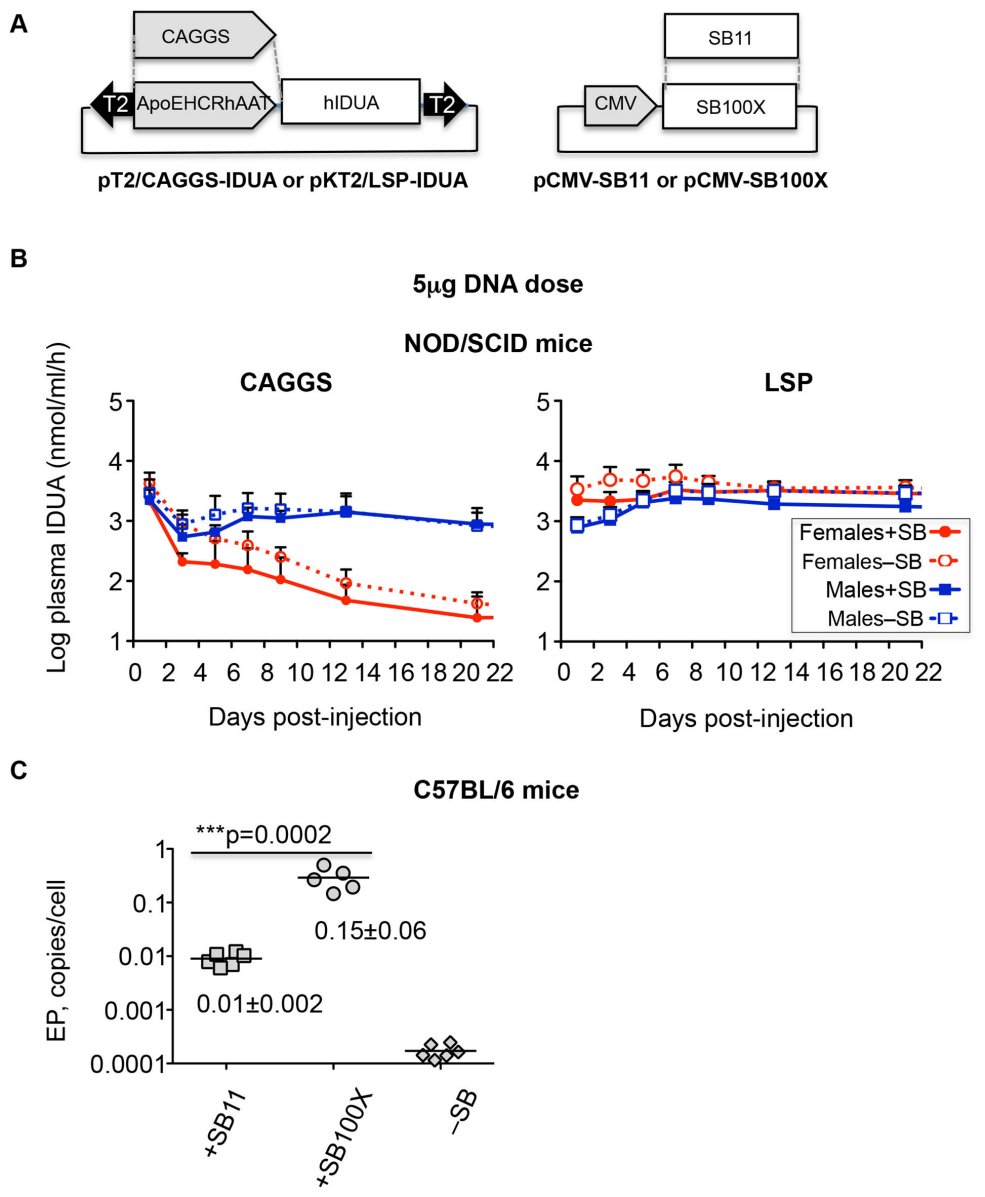
Expression of IDUA from different promoters in NOD/SCID MPS I mice. **A**. Vectors used in this study. The T2 *SB* transposon vectors (inverted arrowheads) contained either a “ubiquitous” CAGGS promoter or the liver-specific promoter (LSP) ApoEHCRhAAT. pKT2/ApoEHCRhAAT-hIDUA and pT2/miniCAGGS-hIDUA have different backbones and the selectable markers, *ka*n and *amp*, respectively. **B**. Kinetics of hIDUA expression from LSP and CAGGS promoters in NOD.129(B6)-Prkdc^scid^IDUA^tm1Clk^/J (NOD/SCID) mice. Symbols: +SB11 females, filled red circles and solid lines; –SB females, open red circles and dotted lines; +SB11 males, filled blue squares and solid lines; –SB males, open blue squares and dotted lines. Each point represents the mean hIDUA activity ± SD, n=6. The CAGGS graph in panel **B**, is from Figure 1d of [8]. **C**. Comparison of transposition efficiency of SB11 and SB100X transposases in C57BL/6 mice by the excision product (EP) assay. 2μg of either pCMV-SB11 or pCMV-SB100X transposase-encoding plasmid was co-delivered with 15μg of pT2/CAGGS-GUSB [7] transposon plasmid. DNA for excision assay was isolated from livers 5 days p.i.

The Sleeping Beauty (SB) transposon system [1] is a non-viral, integrating vector that is an alternative to viral vectors for gene therapy [2–5]. The SB system consists of two parts: the transposon, which is defined by inverted terminal repeats and which carries an expression cassette for the therapeutic gene, and a second expression cassette for the transposase enzyme (Figure 1A). When expressed, the transposase excises the transposon with its cargo from a plasmid and inserts it into the chromosomal DNA of the host. In practice, transposition results in stable integration of the transgene into a vertebrate host genome and prolongs expression of transgenic products such as Factor IX, β-glucuronidase and alpha-L-iduronidase (**IDUA**) [3,6–8] compared to most [9] non-integrating plasmid vectors. 

We have shown in immunodeficient NOD.129(B6)-Prkdc^scid^ *IDUAtm1Clk*/J mice that the SB transposon system is efficacious in gene therapy of murine mucopolysaccharidosis type I (**MPS I**) [8], an inherited systemic disorder caused by IDUA deficiency [10]. However, the NOD/SCID MPS I mouse model (www.jaxmice.com), has drawbacks for studying both transposition and correction of MPS I because of impaired double-strand break repair that severely reduces transposition activity [11] and a shortened lifespan with mild clinical manifestations. However, evaluation of treatment effects in immunocompetent MPS I mice so far has not been feasible due to highly variable expression of IDUA following delivery of SB plasmids [7]. 

In our modified approach, we sought to minimize expression in antigen-presenting cells (APC), increase transposition efficiency by using the hyperactive transposase SB100X, and provide a more effective and less toxic cyclophosphamide regimen by transiently immunosuppressing mice at the time of vector administration. In addition, we aimed at reducing promoter shutdown and gender bias in IDUA expression associated with the CAGGS promoter [8]. As a result, we have attained sustained expression of IDUA in the liver at levels greater than 100-fold wildtype (WT) for one year in at least half of treated mice.

SB-mediated transgene expression is affected by many variables. The efficiency of transposition depends on the transposon and the transposase sequences, which are consistently being improved [2,5]. Since its first use as gene delivery vector [1], the SB transposase has been re-engineered from SB10 [12] through SB11 [13] and other intermediates [14] to the extremely hyperactive SB100X [15]. Likewise, the structure of the original transposon, *T*, has been re-engineered for greater activity, e.g. *T2* [16] and other versions [17]. Besides the transposon system itself and its cargo, factors affecting expression are associated with the properties of expressed proteins, target cells or organs, delivery procedure, etc. [18]. Promoters regulating SB transposase and therapeutic genes can be either ubiquitously expressed or tissue-specific, either prone or resistant to silencing, and drive different levels of transgene expression. The expressed protein can either be restricted to the producer cell, e.g., luciferase [19], or it can escape the cell and be available for recapture by other cells as is typically the case with lysosomal hydrolases such as IDUA [10]. Host-related factors such as genetic background, gender and age can affect expression of the transposed gene. For example, we found that IDUA expression from the CAGGS promoter was silenced in female mice compared to male mice [8]. To gain insights into which variables affecting transgene expression lead to its loss and which facilitate stabilization of IDUA activity at therapeutic level, we examined decay rates in SB-mediated IDUA activity profiles obtained under a variety of conditions. We reasoned that these rates are by and large a reflection of various influences on transgene expression. We found that immune responses are the most important variable to control in order to prevent loss of transgene expression [20].

## Results

### SB transposon vectors support prolonged, high-level expression of human IDUA in immunocompetent mice

We designed a new set of *human* IDUA (hIDUA) expression cassettes to achieve reliable expression by meeting three criteria: 1) minimize expression of transgenic human IDUA in APCs to avoid anti-IDUA immune response; 2) provide high-level IDUA expression in both males and females; and 3) accommodate high-efficiency transposition. To achieve the first goal, we put the hIDUA cDNA under the regulation of a liver-specific promoter (LSP) ApoEHCRhAAT [21–23] instead of the ubiquitous CAGGS we used previously [7,8] (Figure **1A**). Transposons with and without a source of transposase were injected into WT C57BL/6 mice, which, although not IDUA-deficient, are immunogenically comparable to IDUA-deficient mice, because mice mount strong immune responses against human IDUA and innate responses to plasmids. Initial IDUA levels were equally high with both CAGGS and LSP promoters and gender bias was insignificant with the LSP in contrast to the 30-50-fold difference that we observed using the CAGGS promoter (Figure **1B**). Thus, LSP also satisfied our second requirement, i.e., lack of gender bias.

To measure the relative rates of transposition in liver tissue, we used the *excision assay* [24] in which plasmids that have lost the transposon by transposase-mediated excision (called excision products, **EP**) are quantified by qPCR. Comparison of transposition efficiency of SB11 [13] and SB100X [15] transposases by the excision assay in immunocompetent C57BL/6 mice showed that SB100X was about 15-fold more active in liver (Figure **1C**). 

### Expression of hIDUA in C57BL/6 mice over one year

**Figure 2:**
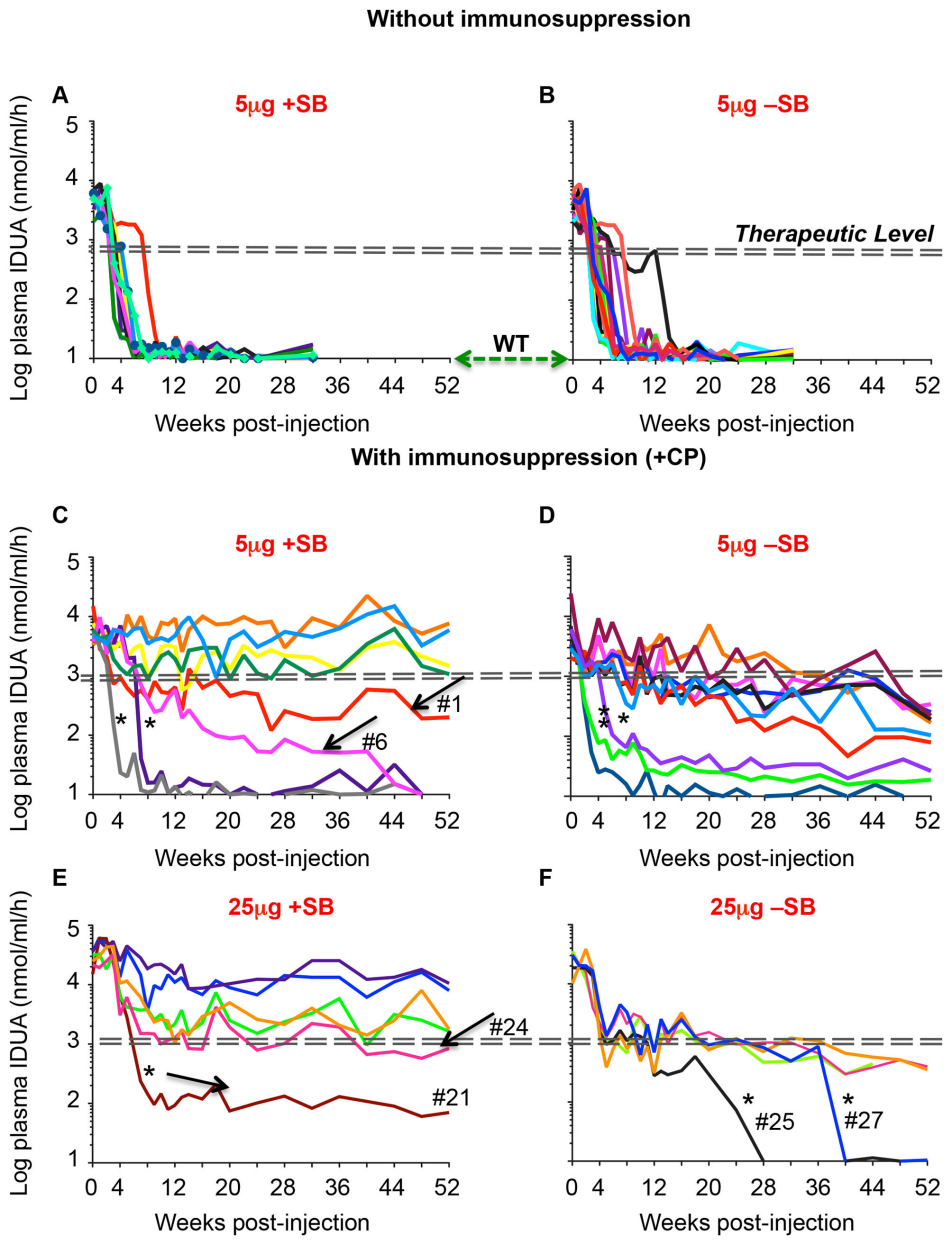
Expression of transgenic hIDUA in C57BL/6 mice. Mice aged 12 weeks were treated with 5μg or 25μg pKT2/LSP-IDUA ± pCMV-SB100X at 5:1 and 10:1 weight ratio, respectively. Plasma IDUA activity in untreated controls (WT) was 10±4 nmol/ml/hr. The dashed double-lines indicate the therapeutic level [8]. Arrows indicate expression profiles of +SB-treated mice with gradual or incomplete loss of IDUA activity. Asterisks indicate rapid decay in CP-immunosuppressed mice. Numbers indicate mouse IDs as listed in Table **1**.

Without immunosuppression, IDUA activity was lost in about 90% of IDUA vector-treated mice by 4-8 weeks post-injection (whether or not SB transposase-encoding plasmid was co-infused (**±SB**, Figures 2A and B). Cyclophosphamide (CP) treatment was used for immunosuppression in a regimen that consisted of four doses (120 mg/kg i.p.), -24, -6, +24 and +48 hrs relative to the hydrodynamic injection. The time-course of IDUA expression within each cohort of mice was not uniform (Figures **2C-F** and Table **1**). In four out of eight female mice treated with 5μg transposon-plasmid and in four out of six mice treated with the 25μg transposon-plasmid (+SB), IDUA activity was sustained for one year at more than 100-fold WT activity (Figures 2C and E), a level that is therapeutically corrective for MPS I mice [8]. Increasing the transposon dose from 5μg to 25μg DNA resulted in an initial approximately 5-fold increase in IDUA activity; however, the latter declined and stabilized by eight weeks post-infusion at levels that were not significantly higher than those attained with the 5μg dose (averages of 456- and 404-fold WT, respectively). In mice that did not receive the transposase plasmid (-SB), IDUA generally declined gradually over a one-year period, ending with an average activity level significantly below the therapeutic range (Figures 2D and F). Some mice appear to have been insufficiently immunosuppressed based on the rapid decline in IDUA expression after 4-36 weeks (Figures 2**C, D** and **F**, asterisks). Examples of gradual decline despite treatment +SB are indicated (Figures 2C and E, 1, 6 and 24, arrows). 

**Table 1:** IDUA expression profiles and activity in plasma *vis-à-vis* transposition and transgene/plasmid maintenance in liver of immunosuppressed C57BL/6 mice one year post-treatment.

**Mouse ID**	**Profile^a^**	**Plasma IDUA**,	**Frequency, DNA copy/cell^b^**		
		**x WT** ^c^	**IDUA**	**EP**	**SB**
5μg**, +SB** (Figure **2C**)					
1	G	20	0.04	bdl^d^	0.02
2	S	768	0.32	0.13	0.11
3	S	148	0.14	0.03	0.03
4	R	1	bdl	bdl	Bdl
5	R	1	bdl	bdl	Bdl
6	R	1	bdl	bdl	bdl
7	S	107	0.04	0.02	0.02
8	S	592	0.08	0.03	0.03
**^e^Mean±SD for S**		**404±330**	**0.15±0.12**	**0.05±0.05**	**0.05±0.04**
5μg**, –SB** (Figure **2D**)					
9	R	1	bdl	bdl	bdl
10	G	17	0.58	bdl	bdl
11	G	34	0.45	bdl	bdl
12	R	2	bdl	bdl	bdl
13	G	25	0.09	bdl	bdl
14	G	19	0.37	bdl	bdl
15	G	8	0.15	bdl	bdl
16	G	22	0.52	bdl	bdl
17	R	3	bdl	bdl	bdl
18	G	10	0.21	bdl	bdl
**^f^Mean±SD for G**		**19.3±8.9**	**0.34±0.19**	**bdl**	**bdl**
25μg**, +SB** (Figure **2E**)					
19	S	178	0.09	0.02	bdl
20	S	801	0.35	0.24	0.03
21	R/S	7	bdl	bdl	bdl
22	S	1051	0.59	0.22	0.03
23	S	164	0.08	0.02	bdl
24	G/S	84	0.07	bdl	bdl
**^e^Mean±SD for S**		**456±440**	**0.28±0.24**	**0.13±0.12**	**0.03**
25μg**, –SB** (Figure **2F**)					
25	G/R	1	bdl	bdl	bdl
26	G	40	0.52	bdl	bdl
27	G/R	1	bdl	bdl	bdl
28	G	35	0.26	bdl	bdl
**^f^Mean±SD for G**		**37.5±3.5**	**0.4±0.2**	**bdl**	**bdl**

a IDUA expression patterns are as defined in the *Results*: **R**-rapid decay; **G**-gradual decay; **S**-stable;**R/S** and **G/R** are hybrid profiles and **G/S** is very slow gradual decay; in **R/S** and **G/S** stabilization not confirmed;

b qPCR determination of copy numbers of DNA sequences of human IDUA and SB100X (transposase) transgenes or excision/repair product plasmids;

c IDUA activity was assayed in plasma; WT in C57BL/6 mice,10 nmol 4MU/ml/h;

d bdl=below detection limit;

e Mean±SD calculated for mice with stabilized expression;

f Mean±SD calculated for mice with gradual expression.

### Maintenance of transgenes and transposition in the liver

**Figure 3:**
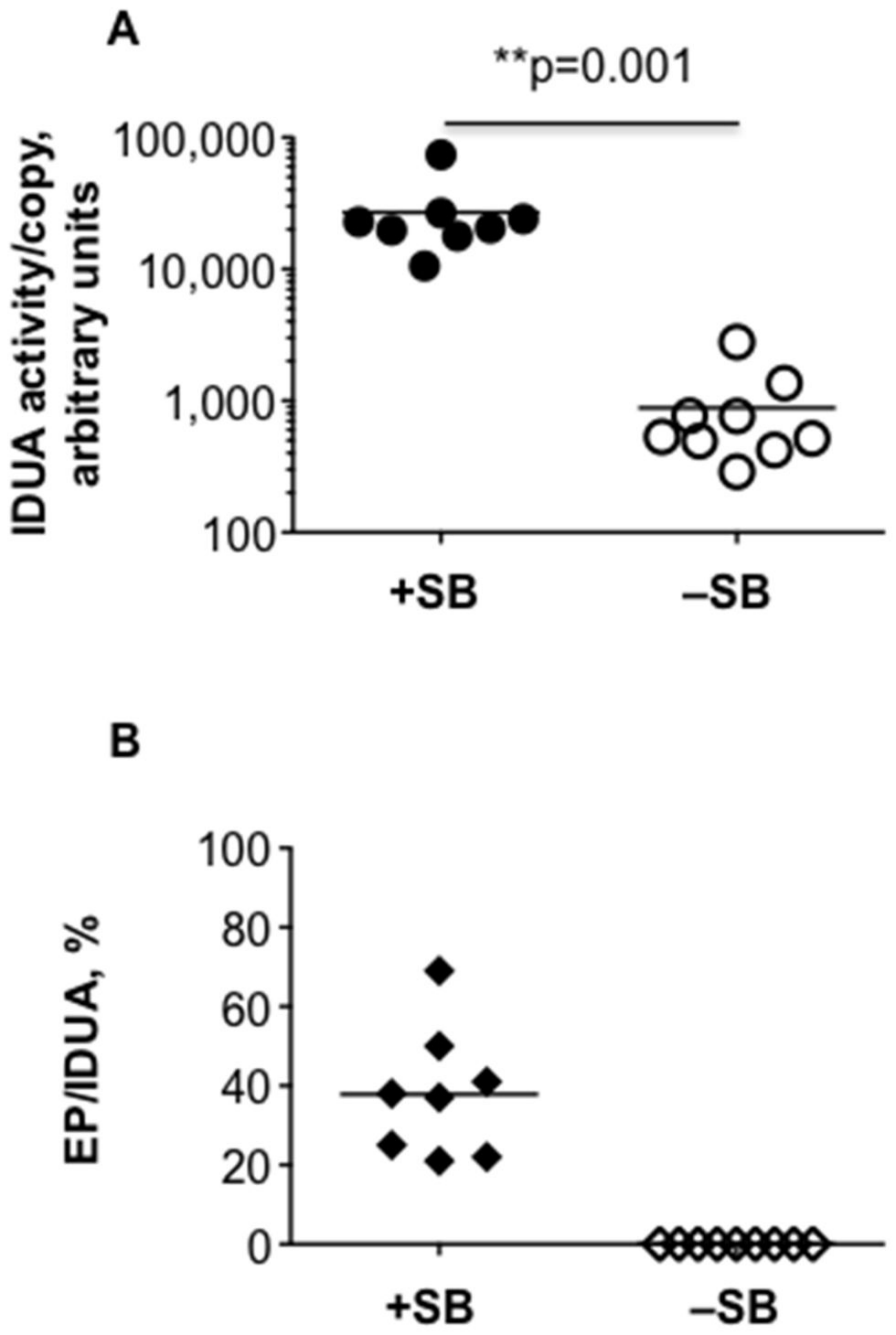
IDUA transgene activity is enhanced when transposition is supported by SB transposase. **A**. Plasma IDUA activities were normalized to IDUA copy per cell number. SB-treated mice with stabilized IDUA activity were compared with mice that were infused with only the transposon plasmids and did not lose IDUA activity in a rapid decay. The IDUA activity/copy number ratio, mean±SD, in arbitrary units, was +SB, 27000±19600 (n=8) and –SB, 900±800 -SB (n=9); **p=0.001. **B**. Transposition efficiency in mice with stabilized expression is calculated as the copy number ratio of pKT2/ApoEHCRhAAT-hIDUA plasmids that underwent transposon excision (EP, copy per cell) to the total number of pKT2/ApoEHCRhAAT-hIDUA (IDUA, copy per cell); mean EP/IDUA±SD =38±16%. Data from mice treated with 5μg and 25μg transposon doses were pooled.

Whenever IDUA activity was rapidly lost (i.e., half-life of less than 3 days), neither transgenes nor plasmid sequences could be detected in samples of mouse liver by qPCR (Table **1**). This suggested that in these cases, cells that initially took up and expressed the transgenic DNA were eliminated from the liver. In contrast, if the expression profile of transgenic IDUA was stable, or declined at a moderate rate, then we could always measure the IDUA transgene level by qPCR. Notably, plasmids that remained after transposition of the transposon cargo, i.e., EP, were detected only in mice with stabilized, high-level IDUA activity (Table **1**). This observation supports our hypothesis that sustained, therapeutic-level transgene expression can be achieved after transposition. EP copy numbers per cell were not significantly different in mice dosed with either 5μg or 25μg of transposon (0.05±0.05 and 0.13±0.12, respectively); the variability in copy number may reflect episomal survival in liver cells one year post-injection. There was no evidence of EPs in mice whose IDUA activities gradually declined over a year (Table **1**), although IDUA transgenes were detected at essentially the same frequency as in those with stabilized activity. Normalizing IDUA activity to IDUA transgene copy number for the non-saturating expression conditions of 5μg of transposon (404/0.15 = 2700 for +SB and 19/0.34 = 57 for –SB, Table 1) indicates that without SB transposase, expression of the transgenes was about 48-fold lower than with transposase (Figure **3A**). Transposition efficiency, reflected by the EP/IDUA ratio for both doses of transposon was 38±2% (n=8) in mice with sustained IDUA expression (Figure **3B**), i.e., about one-third of plasmids that survived as episomes in the liver after one year, had donated a transposon for integration. Our data from the 5μg transposon doses suggest that expression from episomes is about 130-fold (48/0.38) weaker than expression from transposed IDUA expression cassettes, i.e., expression of IDUA genes from episomes is about 1% that from chromosome. 

### Kinetics of loss of IDUA expression

**Figure 4:**
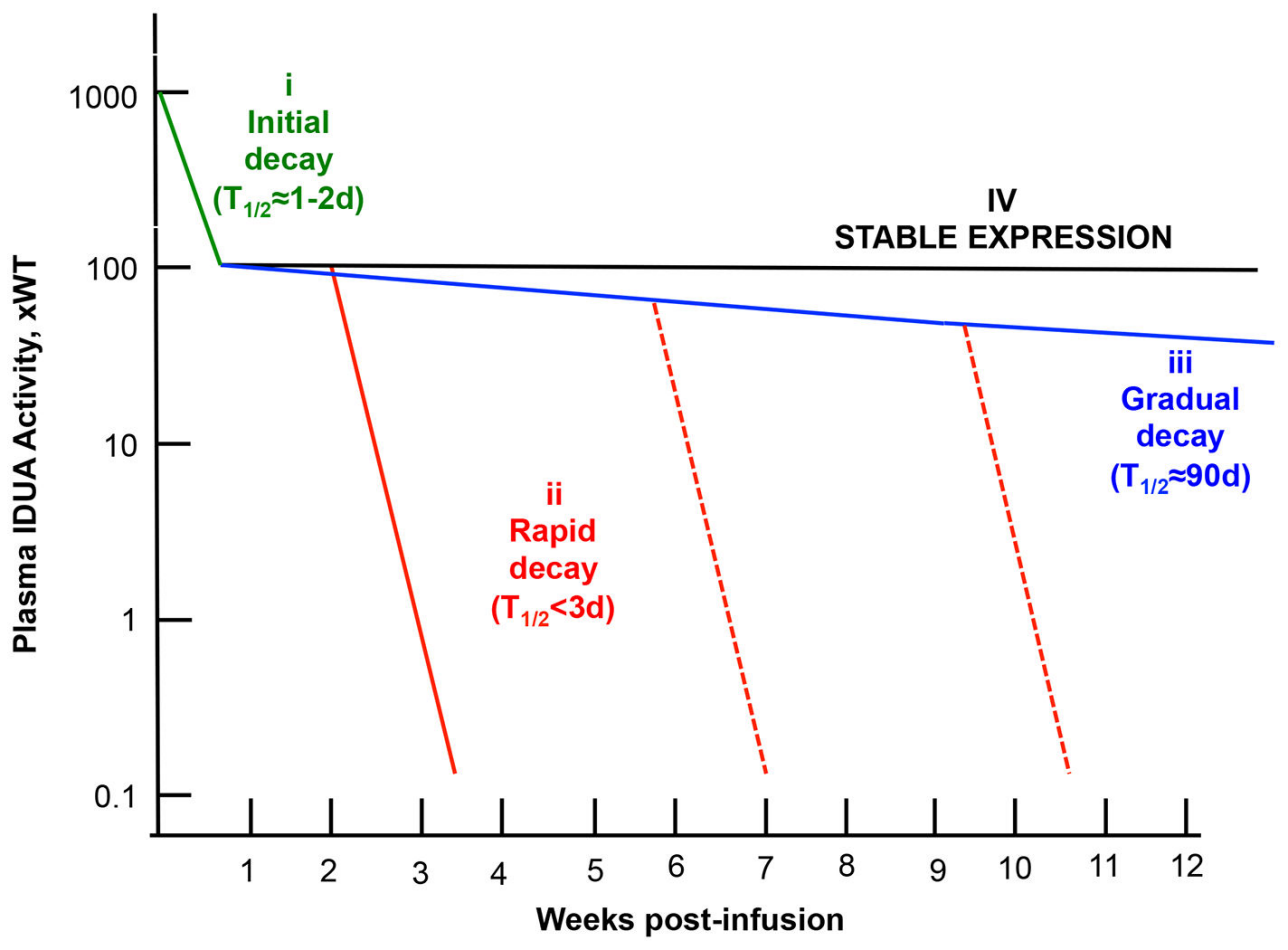
Summary of quantitative evaluation of transgene expression in mice following hydrodynamic delivery to the liver. Three decay rates can account for all of the profiles of expression that we have observed under about 128 different combinations of experimental variables. These decay rates are: *initial* (i), associated with promoter silencing [8], green line; *rapid* (ii), associated with loss of plasmids and transgenes, red line; and *gradual* (iii), blue line. *Stabilized* expression (iv), black line, at levels >100WTdeemed therapeutic [8] was attained only with LSP, transposition using SB 100X transposase and a transient 4-dose immunosuppression with CP administered intraperitoneally around the time of hydrodynamic delivery. We hypothesize that the delayed loss of expression (red dashed lines, mice 25 and 27 in Figure 2F), that has the same half-life of <3 days, is rapid as (ii).

Our inspection of transgene expression profiles under many different experimental conditions indicated that there are several components that account for loss of transgene expression. Quantified decay rates of LSP-IDUA expression from individual mice could be divided into three profiles: *rapid* decay, T_1/2_ =1.6±0.5 days (n=17) calculated from all mice treated without immunosuppression (Figure **4**, red solid line; data in Figures 2A and B); *gradual* decay, T_1/2_= 91±49 days (n=7), typical in CP-immunosuppressed mice that did not receive the transposase-encoding plasmid (Figure **4**, blue line; data in Figure **2D**); and *sustained* expression that was observed only in those immunosuppressed mice that received the SB-transposase-expressing plasmid (Figure **4**, black line; data in Figure **2C**). In those mice that lost expression at unpredictable times (Figure **4**, red dashed lines; mice 25 and 27 indicated by asterisks in Figure **2F**), the half-lives of expression were within the *rapid* decay range observed in animals that were not treated with CP.

*Rapid* decay at its earliest onset occurred between one and two weeks post-infusion, which is consistent with the onset of an adaptive immune response in the absence of immunosuppression by CP. Note that our T_1/2_ values have an inherent imprecision due to the infrequency of plasma IDUA measurements, which prohibits specific identification of the timing of initiation and termination of the decay process. We evaluated IDUA loss-of-expression rates obtained from our earlier studies using a CAGGS-IDUA expression cassette [7,8]. Compared to the LSP expression profiles, with CAGGS there was a gender-biased *initial* decay, steep in females and more *gradual* decay in males, during the first 3 days (Figures **1B** and [8]). Thus, despite differences between CAGGS and LSP-expression due to gender bias and promoter silencing associated with CAGGS, the rates of final *elimination* of IDUA expression (T_1/2_=1-2 days) were similar for the LSP and CAGGS promoters, as was clearance of transgenes and excision products in those mice where all activity was lost. 

## Discussion

Achieving effective, life-long gene therapy is daunting because many variables can affect transgene expression. Such is the case with SB-mediated gene therapy for MPS I, where the goal is sustained, high-level IDUA expression in the liver. 

In our previous work with the NOD/SCID MPS I model, IDUA expression profiles within a treatment group were consistent whereas high variability and inconsistency of expression were evident in immunocompetent MPS I mice even when immunosuppressed. This study, which employed delivery of immunogenic *human* IDUA-encoding transposons to immunocompetent mice, is a good model in which to examine the variable kinetics of transgene expression that one would expect in immunocompetent IDUA-deficient animals. Our achievement of sustained, high-level IDUA expression in 50% of C57BL/6 mice (Figures 2C and E) mimics what we have observed when treating MPS I mice. Our best results were obtained using the complete transposon system +SB100X transposase, a liver-specific promoter, and a single, four-dose regimen of CP immunosuppression around the time of transgene delivery. While the two former modifications to minimize immune responses are obvious, demonstration of SB-mediated effects on expression over one year has not been reported. That higher levels of SB activity were indeed attained is shown by quantitative measurements of excision circles (Figure **1C**), and the levels of plasma IDUA following SB transposition (e.g., Figure **2C**) are higher than those we have previously reported [7,8]. Our data suggest that transposition of the transgenic expression cassette into chromatin is necessary for long-term activity following plasmid delivery to the liver. However, stable transgene expression has been reported for minicircles [9,25–27] and mini-intronic plasmids [28], presumably because they either lack sequences that lead to transcriptional silencing [9,27] or are contained in an intron within the eukaryotic expression cassette [28]. Notably, in effectively immunosuppressed C57BL/6 mice, LSP-driven expression from episomes might continue for months, although we have never observed stabilized expression from episomes at >100 WT and the resulting episomal IDUA expression levels after one year never reached those deemed therapeutic for MPS I [8]. These observations are consistent with our calculations that expression from whole plasmids is about 1% that from transposed expression cassettes. They are also similar to what we saw upon Cre recombinase-induced inactivation of non-transposed erythropoietin transposons [29].

Prolonged persistence of low-level transgene expression from episomes may explain the absence of a clear-cut distinction in transgene activity between chromosomally integrated compared to episomal transposons in shorter-term experiments. By extending our studies out to one year, the long-term effects of using the complete SB system to achieve chromosomal integration of the IDUA expression cassette became obvious. Transposon doses of 5μg and 25μg yielded comparably high levels of stabilized transgenic IDUA activities (Figure 2C and E, Table **1**). Although the increase of transposon+transposase dose from 5+1μg to 25+2.5μg, respectively, resulted in the expected approximately 5-fold increase in day-1 IDUA activity, there was a decline in average transgene expression in the 25+2.5μg dosed mice that ranged from 5 to 20-fold from peak activity at stabilization around 8 weeks p.i. (ibid). We interpret the gradual initial declines in +SB and –SB mice dosed with 25μg transposon-plasmid as being due to innate immune responses to a higher concentration of plasmid sequences, which can trigger transcriptional repression [9,25,27]. Thus, “more may be less” in the case of loading hepatocytes with transgenic constructs that remain as episomes. 

Comparison of expression levels indicates the importance of carefully choosing the transgene promoter. IDUA activity from LSP was at therapeutic levels without the drawback of the initial fast shutdown of expression from CAGGS in females, which occured in both C57BL/6 and NOD/SCID mice [8] (Figures **1B** and **4**). However, the liver-specific promoter alone is not a solution against loss of expression, even with transposition. Although liver-specific (i.e., hepatocyte-specific) promoters minimize expression from APCs, overexpressed IDUA may not evade a robust immune system. Thus, while LSP-IDUA expression is not prone to silencing, LSP does not rescue expression from elimination by adaptive immune responses. Without immunosuppression, *rapid* decay, (Figures 2A and B) occurred at a time consistent with the onset of the adaptive immune response, 7 to 14 days post-infusion. Osborn et al. [28] demonstrated the presence of anti-IDUA IgG antibodies in the sera of mice treated with minicircle-encoded human IDUA without costimulatory blockade. However, it appears that cell-mediated responses are responsible for ultimately eliminating IDUA expression in both their experiments and in ours. These observations parallel ours in non-immunosuppressed mice that received CAGGS-IDUA; CTL-mediated immune responses removed transduced hepatocytes throughout the liver [7]. In this study, such rapid loss was evaded in over 70% of the mice treated with LSP-IDUA and transient CP regimen. 

There are two plausible explanations for complete rapid loss of transgene expression in mice that were immunosuppressed: 1) some mice are resistant to immunosuppression by CP and 2) some intraperitoneal injections failed due to inadvertent placement of the injection other than intraperitoneal cavity [30,31]. Partial failure of immunosuppression with a 4-dose regimen could explain some of the outlier profiles of IDUA expression that we observed (Figures 2C and E, arrows). If loss of activity in the first six weeks in the 5μg and 25μg transposon-dosed groups was due to failed immunosuppression, then nearly 75% (8/11) of the truly immunosuppressed mice had stabilized expression of IDUA at therapeutic levels. If this is the case, our data suggest that improvement of immunosuppression will lead to sustained therapeutic effects in most mice. 

In conclusion, in this and our previous studies [7,8,32] we have examined the levels of transgene expression for up to one year in transposon-injected mice under about 128 different combinations of SB transposases, transgene promoters, mouse genetic backgrounds, mouse gender, and cyclophosphamide immunosuppression. Broadly, two basic phenomena lead to the loss of transgene expression – presumably, transcriptional silencing and adaptive immune responses. Our quantitative analyses allowed us to discern the different kinetics underlying loss of transgene expression and better interpret experimental results. These causes can be divided into the ones that are feasible to control (e.g., choice of promoter, immunosuppression protocol, plasmid dose) and those that are poorly understood. We conclude that despite what looks like a large variation in patterns of transgene expression, many combinations of factors affecting transgene expression lead to only a limited number of post-treatment scenarios (Figure **4**). The SB system is capable of providing effective gene therapy in mice, but improved methods of immunosuppression are needed to increase the percentage of treated animals that do not lose transgene expression. 

## Materials and Methods

### Ethics statement

Mice were housed under specific pathogen-free conditions in AAALAC-accredited facilities. The Institutional Animal Care and Use Committee of the University of Minnesota approved all animal studies, protocol #1202A09921. Hydrodynamic injections were performed while the animals were under light anaesthesia to minimize suffering. 

### Plasmids

The source of the liver-specific promoter (LSP) ApoEHCRhAAT was pAAV-hFIX16 [23], a gift of Dr. Mark Kay (Stanford University, USA). The SB transposon pKT2/ApoEHCRhAAT-IDUA was engineered by subcloning an *Eco*RI-*Eco*RI fragment containing the full-length human α-L-iduronidase (*hIDUA*) cDNA into a unique *Eco*RI site of the L212-pKT2-ApoE-hAAT-BGintron plasmid. The ApoE-hAAT-BG intron was constructed by PCR amplifying the β-globin non-coding exon/mini intron from the mini-CAGGS vector and splicing it downstream of the ApoE-hAAT promoter. The PCR product was then TOPO-cloned (Invitrogen, Grand Island, NY, USA) and verified by sequencing. Subsequently, the ApoE-hAAT-BG was subcloned into the pKT2 vector containing the rabbit β-globin poly (A). The resulting plasmid, about 6.1 Kbp, had a kanamycin resistance gene, T2 inverted terminal repeats [16], the rabbit β-globin intron upstream and the rabbit β-globin polyadenylation site downstream the IDUA sequence, the latter was previously described [8]. The plasmid for transposase expression pCMV(CAT)T7-SB100, 4752 bp [15], was a gift from Dr. Zoltan Ivics (Max Delbruck Centre, Berlin, Germany). We refer to it in the text as pCMV-SB100X. As a negative control for transposition, the transposon plasmid was co-delivered with the pBluescript plasmid (Stratagene, La Jolla, CA, USA). 

### Animals and injections

C57BL/6 mice were purchased from Charles River (Wilmington, MA, USA) and housed under specific pathogen-free conditions in AAALAC-accredited facilities. Plasmids were prepared commercially (Aldevron, Fargo ND) and injected into mice using the hydrodynamics-based procedure as described [33]. Mice, in which complete dose failed to be injected or the injection time exceeded 7 sec, were eliminated from the study. Blood was collected at selected times by facial-vein phlebotomy without anesthesia. Animals were euthanized by carbon dioxide inhalation and perfused with saline. Livers were harvested and preserved for analyses as specified below. Cyclophosphamide (CP) at the dose of 120 mg/kg was injected intraperitoneally 24 h and 6 h before and 24 and 48 h after the hydrodynamic plasmid infusion. 

### IDUA enzyme assay

Liver and plasma specimens were stored at -80°C. Whole-liver specimens were pulverized by freezing in liquid nitrogen and grinding with mortar and pestle. Portions of each sample were extracted to assay for IDUA and plasmid excision product copy number by qPCR. IDUA activity was measured in plasma using the fluorometric assay with 4-MU-α-L-iduronide (Glycosynth, England) as a substrate, as described [34]. 

### PCR analyses and copy number determinations

DNA was isolated from about 50 mg pulverized whole-liver specimens by phenol-chloroform extraction (www.molecularcloning.com). DNA copy number was determined by real-time PCR using an iCycler (Eppendorf), as previously described [7,8]. The mouse glyceraldehyde-3-phosphate dehydrogenase (GAPDH) sequence, which served as an internal control of genomic DNA content, was amplified in a separate reaction. Standard curves for GAPDH, IDUA and SB genes and plasmid “excision products” (EP) were obtained as described (ibid). The mockEP used for preparation of EP standard curve was derived from the pKT2/ APOe-hAAT-BGintron plasmid in which the transposon was deleted between *Pvu*II and *Dra*I sites. The PCR primers for the excision assay were: FP-5’GCCTCGACGTTTCCCGTTGA and RP: 5’-GCGAGGAAGCGGAACAGATT. The primers to amplify both SB11 and SB100X were obtained from Dr. Vincent Keng (then at the University of Minnesota):

FP: 5’-ATGGGAAAATCAAAAGAAATCAGCC and 

RP: 5’-CGCACCAAAGTACGTTCATCTCTA.

### Statistical analysis

Data were analyzed using GraphPad Prizm 4.0 software (GraphPad Software Inc., San Diego, CA). The significance of differences between groups was determined based on exact two-tailed *p* values obtained with the Welch’s t-test. A value of *p* < 0.05 was considered statistically significant. 
